# NK cell–intrinsic FcεRIγ limits CD8^+^ T-cell expansion and thereby turns an acute into a chronic viral infection

**DOI:** 10.1371/journal.ppat.1007797

**Published:** 2019-06-20

**Authors:** Vikas Duhan, Thamer A. Hamdan, Haifeng C. Xu, Prashant Shinde, Hilal Bhat, Fanghui Li, Yahya Al-Matary, Dieter Häussinger, Judith Bezgovsek, Sarah-Kim Friedrich, Cornelia Hardt, Philipp A. Lang, Karl S. Lang

**Affiliations:** 1 Institute of Immunology, Medical Faculty, University of Duisburg-Essen, Essen, Germany; 2 Department of Molecular Medicine II, Medical Faculty, Heinrich Heine University, Düsseldorf, Germany; 3 Department of Hematology, West German Cancer Center, University Hospital Essen, University of Duisburg-Essen, Essen, Germany; 4 Department of Gastroenterology, Hepatology, and Infectious Diseases, Heinrich‐Heine‐University, Düsseldorf, Germany; University of Massachusetts Medical School, UNITED STATES

## Abstract

During viral infection, tight regulation of CD8^+^ T-cell functions determines the outcome of the disease. Recently, others and we determined that the natural killer (NK) cells kill hyperproliferative CD8^+^ T cells in the context of viral infection, but molecules that are involved in shaping the regulatory capability of NK cells remain virtually unknown. Here we used mice lacking the Fc-receptor common gamma chain (FcRγ, FcεRIγ, *Fcer1g*^*–/–*^ mice) to determine the role of Fc-receptor and NK-receptor signaling in the process of CD8^+^ T-cell regulation. We found that the lack of FcRγ on NK cells limits their ability to restrain virus-specific CD8^+^ T cells and that the lack of FcRγ in *Fcer1g*^*–/–*^ mice leads to enhanced CD8^+^ T-cell responses and rapid control of the chronic docile strain of the lymphocytic choriomeningitis virus (LCMV). Mechanistically, FcRγ stabilized the expression of NKp46 but not that of other killer cell–activating receptors on NK cells. Although FcRγ did not influence the development or activation of NK cell during LCMV infection, it specifically limited their ability to modulate CD8^+^ T-cell functions. In conclusion, we determined that FcRγ plays an important role in regulating CD8^+^ T-cell functions during chronic LCMV infection.

## Author summary

FcRγ is a signaling molecule for Fc receptors and NK cell killer activating receptor (KAR) complex. FcRγ is highly expressed by NK cells and involved in NK cell activity. NK cells are widely defined to regulate the expansion of T cells. Here using chronic LCMV model, we described the role of FcRγ in NK cell mediated shaping of CD8^+^ T cell response and viral control. We observed that FcRγ does not affect the early activity of NK cells which is mainly innate immune cytokines driven, but rather the specific activation due to NKp46 inadequacy. We detected that FcRγ stabilizes NKp46 protein by preventing it from proteasomal degradation. Due to lack of NKp46 expression in absence of FcRγ, we observed strong CD8^+^ T cell response and faster viral clearance during chronic LCMV infection. These data demonstrate that FcRγ is crucial for specific activity of NK cells for regulation of CD8^+^ T cell response during viral infection.

## Introduction

CD8^+^ T cells are key antiviral effector cells during infection with persistence-prone viruses, such as hepatitis B virus (HBV), hepatitis C virus (HCV), and human immunodeficiency virus (HIV) [1]. Several host factors promote tight regulation of CD8^+^ T-cell functions, thereby modulating the outcome of chronic viral infection. The role of co-stimulatory and inhibitory molecules that are expressed on myeloid cell types and T cells and that modulate CD8^+^ T-cell functions has been intensively studied, and these studies have led to the development of several immune-modulating drugs [2]. CD8^+^ T-cell functions are also modulated by suppressor lymphocytes such as CD4^+^ regulatory T cells or natural killer (NK) cells [3].

Recently, we and others have determined that NK cells play a crucial role in modulating CD8^+^ T-cell functions [4–6]. NK cells can be activated by type I interferon (IFN-I) during viral infection [7] and exhibit cytotoxicity triggered by the molecule perforin and granzymes [8]. During chronic infection with the lymphocytic choriomeningitis virus (LCMV), NK cells recognize hyperproliferative CD8^+^ T cells and kill them in a perforin-dependent manner [9, 10]. Although IFN-I signaling in CD8^+^ T cells is one regulating factor that renders CD8^+^ T cells highly sensitive to NK cell–mediated killing, factors modulating the regulatory functions of NK cells are mainly unknown.

A milieu of inhibitory, co-stimulatory, and activating receptors orchestrates the killing function of NK cells; these receptors include Fc receptors and various killer cell–activating receptors (KARs) [5]. Among the activating NK cell receptors, NKp46 is a unique member of the natural cytotoxicity receptor (NCR) family, which also includes NKp30 and NKp44. NKp46 (CD335) has the only mouse ortholog, which is encoded by *NCR1* gene, and is the main activating receptor expressed by both resting and activated NK cells [11]. NKp46 directly recognizes the hemagglutinin (HA) proteins of influenza viruses [12] and of other viruses such as poxviruses and the Newcastle disease virus [13]. Recently, some non-viral ligands have been elucidated, such as complement factor P and surface protein on healthy pancreatic β cells. Although the identities of the cellular ligands of NKp46 are still elusive, the putative hypothesis is that NKp46 is involved in an array of immunological activities [14, 15].

The receptors of the Fc portion of immunoglobulins (Fc receptors, FcRs) are ubiquitously found on a variety of cell types of the immune system with versatile functions and belong to the large immunoglobulin superfamily and are type I transmembrane glycoproteins [16]. The γ (gamma) subunit of immunoglobulin Fc receptor (FcRγ) is essential homodimeric or heterodimeric part for different Fc receptors. FcRγ is an intracellular common gamma chain signaling molecule for several receptors including FcεRI, FcγRI, FcγRIII (CD16), FcαRI, Dectin-1&2, LMIR8, MARR-I&II, OSCAR, TREM, PIR-A, active γδTCR, IL-3R and NKp46 [17, 18]. FcRγ contains immunoreceptor tyrosine-based activation motifs (ITAMs) in cytoplasmic tail and upon receptor ligation induces activation of Src and Syk family kinase signalling [18]. CD16 is an activating receptor that is highly expressed on human NK cells [19] and modestly on murine NK cells [20]. FcRγ is called FcεRIγ because FcRγ was first noticed as the third subunit of FcεRIγ [21].

FcRγ is associated with NKp46 in transmembrane region and among the molecules which play a prominent role in NK cell activation signaling. Moreover, the transmembrane part of FcRγ is disulfide bonded to CD3ζ (encoded by CD247 gene) and both of them contain ITAMs in cytoplasmic region which initiate signaling downstream to NKp46 [22, 23]. A previous study in healthy individuals revealed a novel subset of human NK cells which are deficient in FcRγ with reduced expression in NKp46 and exhibited poor reactivity toward tumor targets [24]. Nevertheless, the impact of FcRγ during chronic viral infection remains to be investigated.

The study reported here determined that FcRγ stabilizes NKp46 protein and protects it from proteasomal degradation. Lack of FcRγ leads to a defect in NKp46 expression on NK cells, and hence compromising their activity to target cells. We found that FcRγ is the main player in the regulatory function of NK cells during chronic LCMV infection. Therefore, the lack of FcRγ in *Fcer1g*^*–/–*^ mice leads to rapid control of chronic LCMV infection.

## Results

### FcεRIγ is an important contributor to NK cell–mediated regulation of virus-specific CD8^+^ T cells

**Fig 1:**
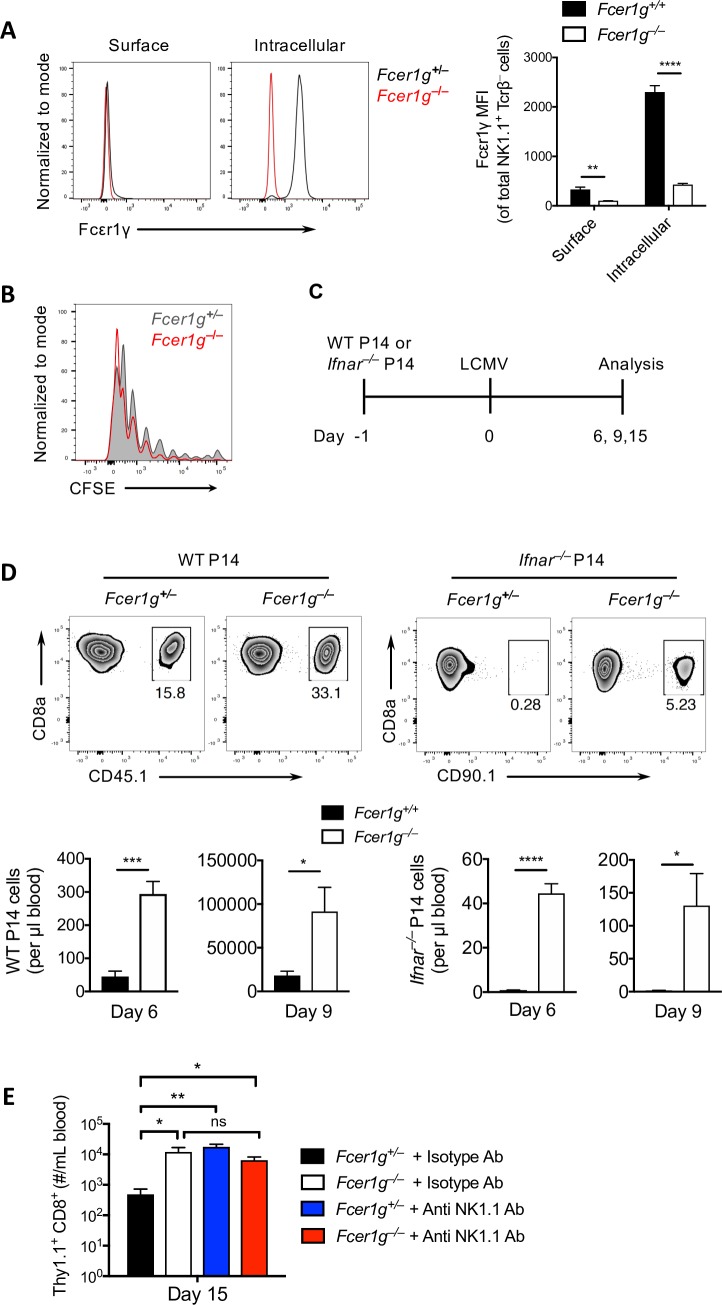
FcεRIγ is an important contributor to NK cell–mediated regulation of virus-specific CD8^+^ T cells. (A) Surface and intracellular FcεRIγ expression by natural killer (NK) cells from spleens of *Fcer1g*^+/–^ and *Fcer1g*^*–/–*^ mice that had been infected intravenously (i.v.) with 2 × 10^4^ plaque-forming units (PFU) of the Docile strain of the lymphocytic choriomeningitis virus (LCMV-Docile). Cells were analyzed 48 hours after infection (n = 4). (B) The histogram shown depicts the proliferation capacity of P14 cells in spleen represented as CFSE dilution in *Fcer1g*^+/–^ and *Fcer1g*^–/–^ mice. 10^7^ CFSE-labelled splenocytes from P14 x CD45.1 mice were adoptively transferred into *Fcer1g*^+/–^ and *Fcer1g*^–/–^ mice. After one day mice were i.v. infected with 200 PFU of LCMV-WE strain and the CFSE dilution was assessed in spleen at day 4 (n = 4). (C) Schematic of the experimental setup. (D) Splenocytes (10^4^) from WT P14 or *Ifnar*^–/–^ x P14 mice were adoptively transferred into *Fcer1g*^*+/–*^ or *Fcer1g*^*–/–*^ mice one day earlier, then the mice were i.v. infected with 2 × 10^4^ PFU of LCMV-Docile. In the upper panel, shown are representative histograms for the frequencies of WT P14 or *Ifnar*^*–/–*^ P14 cells at day 6 post-infection. In the lower panel, the bar graph represents total number of transferred WT P14 or *Ifnar*^*–/–*^ P14 cells in the blood at the indicated days after infection (n = 4). (E) 10^4^ splenocytes from P14 × *Ifnar*^*–/–*^ mice were transferred into *Fcer1g*^+/–^ or *Fcer1g*^–/–^ mice that had been treated with isotype antibody or anti NK1.1 antibody at day 3 and 1 before i.v infection with 2 × 10^4^ PFU of LCMV-Docile. The graph shows the total number of transferred P14 cells in blood at day 15 post-infection (n = 3–4). Data are shown as mean ± SEM. Significant differences between the two groups were detected by unpaired two-tailed *t*-tests and are indicated as follows: ns, not significant; * *p<*0.05; ** *p*<0. 01; *** *p*<0.001; **** *p*<0.0001.

We and others have found that NK cells play an indispensable role in shaping the antiviral CD8^+^ T-cell response during LCMV infection [5, 9, 10]. The molecular mechanisms by which NK cells orchestrate this regulatory function remain unknown. IFN-I plays a cardinal role in this regulatory function of NK cells because CD8^+^ T cell–intrinsic IFN-I signaling protects CD8^+^ T cells from NK cell–mediated cytotoxicity [9, 10]. This function is seen in interferon-α/β receptor deficient (*Ifnar*^*–/–*^) CD8^+^ T cells, which are highly susceptible to NK cell regulation. To determine whether FcRγ is involved in the regulatory function of NK cells, we first determined the expression of FcRγ by NK cells. NK cells exhibited higher expression of intracellular FcRγ but minimal surface expression of FcRγ (Fig 1A).To determine the impact of FcRγ molecule on the proliferative capacity of virus-specific CD8^+^ T cells in *Fcer1g* –sufficient /deficient mice, we adoptively transferred the CFSE labelled LCMV–specific transgenic CD8^+^ T cells (P14 cells) into WT and *Fcer1g*^–/–^ mice and infected the mice with LCMV-WE strain. P14 cells were found to have negligible to slight enhanced proliferative capacity in the spleens of *Fcer1g*^*–/–*^ mice as compared to WT as indicated by CFSE dilution (Fig 1B). To study the functions of FcRγ in the NK cell regulation of CD8^+^ T-cell activity including their role in IFN-I mediated protection of T cells response, we transferred either wild-type (WT) P14 cells (WT P14) or P14 cells that lack the receptor for IFN-I (*Ifnar*^*–/–*^ × P14) into *Fcer1g*^+/–^ and *Fcer1g*^*–/–*^ mice and then infected the mice with LCMV-Docile (Fig 1C). On day 6 and 9 upon infection, we found that the number of WT P14 cells was specifically reduced in *Fcer1g*^+/–^ but not in *Fcer1g*^–/–^ mice (Fig 1D, left panel), indicating the robust expansion of virus-specific CD8^+^T cells in the mice that are devoid of FcRγ. The number of *Ifnar*^*–/–*^ P14 cells, which are the main target of NK cells was vanished in *Fcer1g*^+/–^ mice and partially rescued in *Fcer1g*^–/–^ mice as compared to that of WT P14 transfer after infection with LCMV-Docile (Fig 1D, right panel). A finding claiming that WT P14 and *Ifnar*^*–/–*^ P14 cells show augmented expansion in *Fcer1g*^*–/–*^ mice. To further know if this phenotype is due to cytotoxic activity of NK cells, we depleted NK cells in WT or *Fcer1g*^–/–^ mice and analysed the expansion of *Ifnar*^*–/–*^ P14 cells. Consistently, *Fcer1g*^–/–^ mice but not WT mice exhibited higher CD8^+^ T-cell expansion similar to that found in NK cell–depleted WT or *Fcer1g*^–/–^ mice (Fig 1E). From these findings we conclude that FcRγ is an important mediator of NK cell regulation during LCMV infection.

### FcεRIγ has no impact on NK cell activation but does affect the expression of NKp46

**Fig 2:**
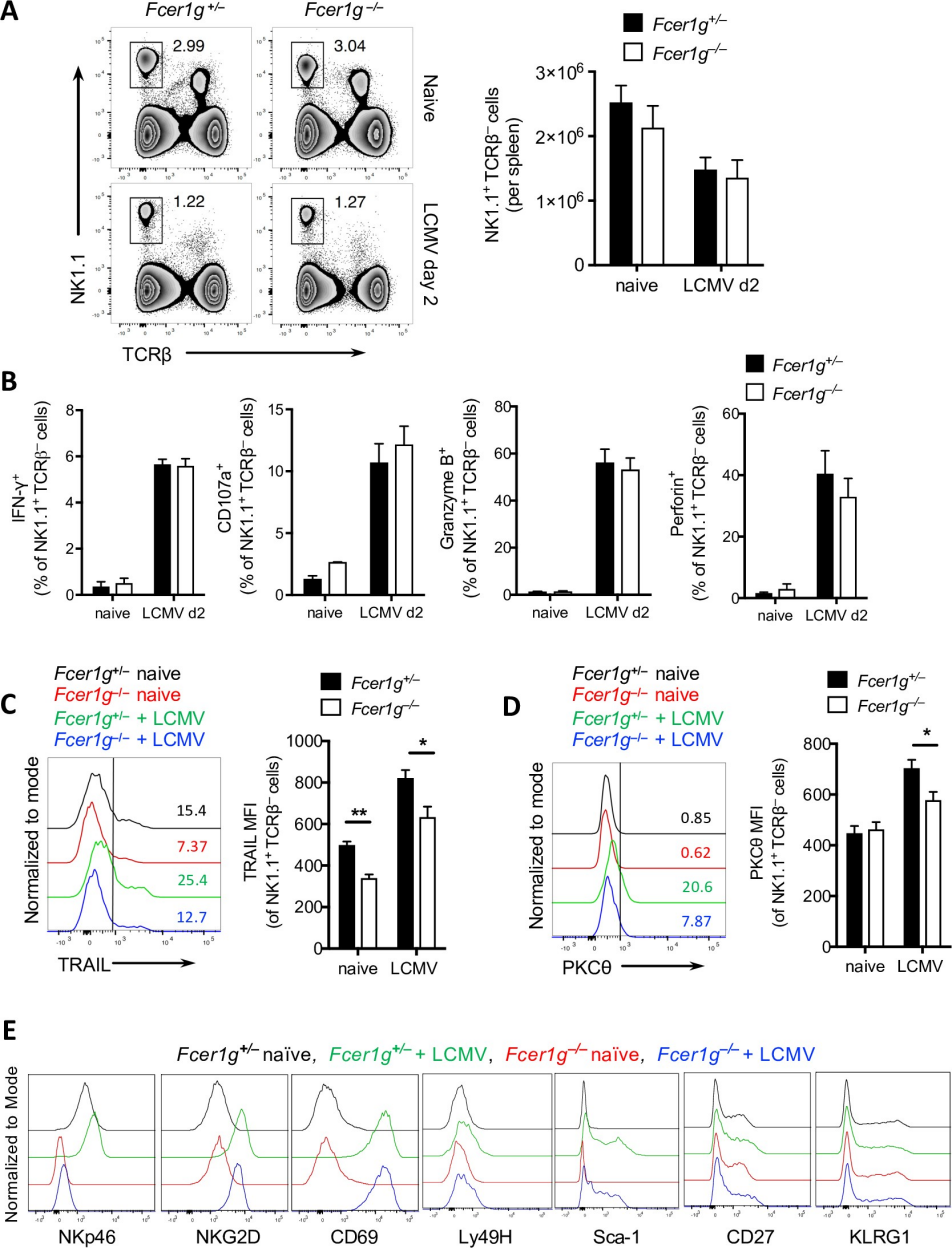
FcεRIγ has no impact on NK cell activation but does affect the expression of NKp46. *Fcer1g*^+/–^ and *Fcer1g*^–/–^ mice were left untreated or were infected i.v. with 2 x 10^4^ PFU of LCMV-Docile. Mice were put to death on day 2 (d2) after infection and NK cells in the spleen were analyzed for various markers by flow cytometry. (A) Representative fluorescence-activated cell sorting (FACS) plots for the frequencies of NK cells (left panel). The bar graph in right panel shows total number of NK cells in naïve and LCMV-Docile infected mice (n = 4). (B) Frequency of various markers in intracellularly stained NK cells from naïve and LCMV infected mice (n = 3–5). Data are pooled from two independent experiments. (C) Surface expression of TRAIL on splenic NK cells from naïve and LCMV-infected mice (n = 3–4). (D) Intracellular staining of PKC-θ on splenic NK cells from naïve and LCMV-infected mice (n = 3–4). (E) Representative histograms for various cell surface markers on NK cells from naïve and LCMV-infected mice (n = 3–4). Experimental data are representative of three independent experiments. Data are shown as mean ± SEM. Significant differences between the two groups were detected by unpaired two-tailed *t*-tests and are indicated as follows: * *p<*0.05; ** *p*<0. 01.

Next, we focused on the mechanism by which FcRγ affects the regulatory function of NK cells. NK cells in naive and LCMV-infected *Fcer1g*^+/–^ and *Fcer1g*^–/–^ mice were equivocal in frequencies and numbers (Fig 2A). Activation of NK cells depends on cytokine signaling and activating receptors [25, 26]. Next, we focused to determine how innate immune activation after viral infection affects the activity of NK cells. We found similar levels of NK cell effector molecules such as IFN-γ, granzyme B and perforin during early LCMV infection in *Fcer1g*^+/–^ and *Fcer1g*^–/–^ mice (Fig 2B). Therefore, we concluded that the development and activation of NK cells do not explain the lack of NK cell regulatory functions. Nevertheless, the killing function of NK cells may be impaired in *Fcer1g*^–/–^ mice. Expression of TNF-related apoptosis-inducing ligand (TRAIL), which directly induces cell death [27] was specifically reduced in the absence of FcRγ (Fig 2C). The expression of protein kinase C theta (PKC-θ), which is activated by NK cell–activating receptors [28] and IFN-I signaling [29], and is required for the production of cytotoxic mediators was found to be reduced in *Fcer1g*^–/–^ mice upon LCMV infection (Fig 2D).

Next, we analyzed the NK cell activation associated known molecules that are linked or not linked with FcRγ. Although, we detected no differences in the expression of NKG2D, Sca-1, Ly49H, CD69, CD27, and KLRG1 between *Fcer1g*^+/–^ and *Fcer1g*^–/–^ NK cells but found that NKp46 expression was absent in *Fcer1g*^–/–^ NK cells (Fig 2E). These findings demonstrate that the lack of FcRγ in NK cells result in missing of NKp46 expression and reduced TRAIL expression which might be the reason for restricted regulatory capability of *Fcer1g*^*–/–*^ NK cells.

### FcεRIγ stabilizes NKp46 on NK cells

**Fig 3:**
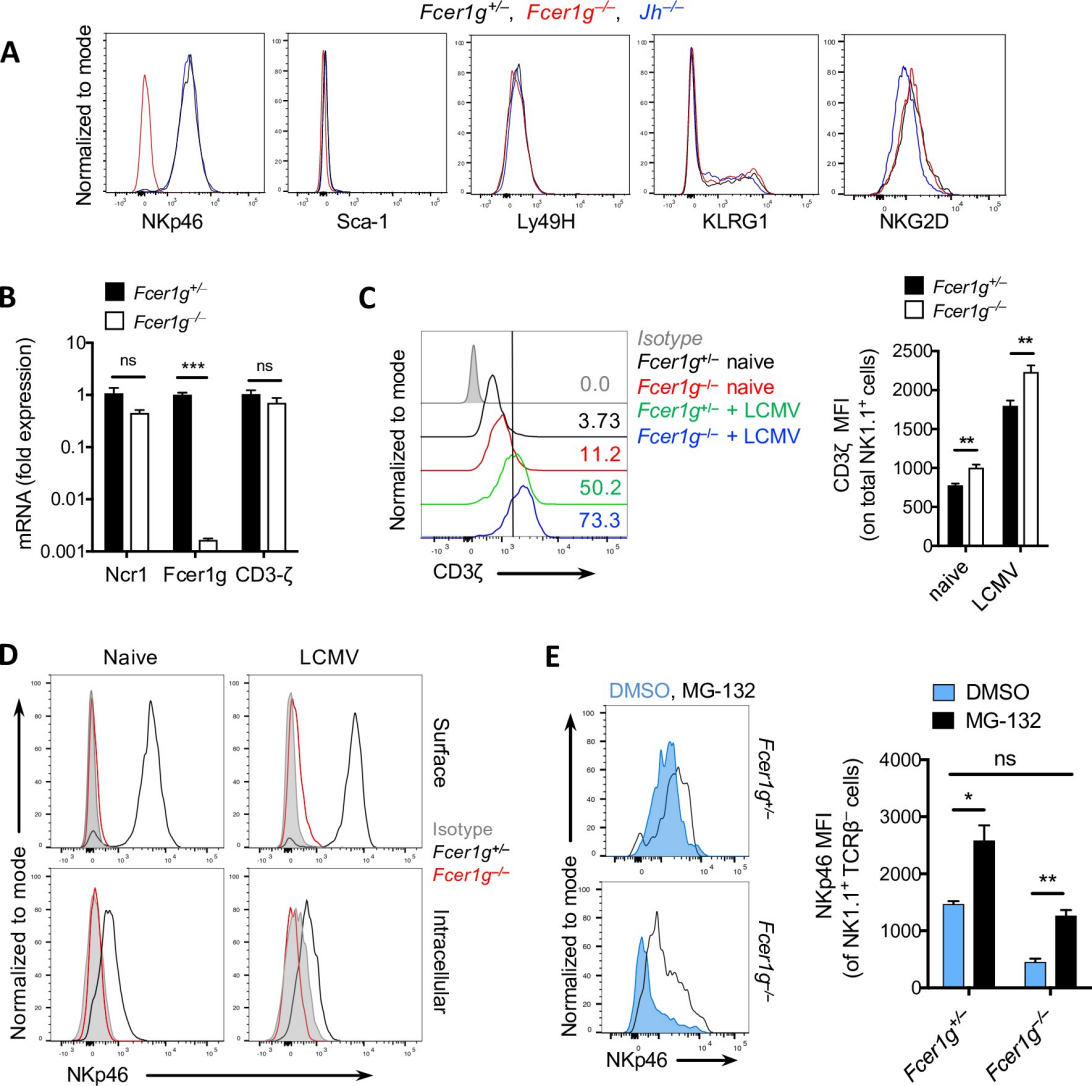
FcεRIγ stabilizes NKp46 on NK cells. (A) Surface analysis of various markers on splenic NK cells of naïve *Fcer1g*^+/–^, *Fcer1g*^–/–^, and *Jh*^–/–^ mice (n = 4). Shown histogram is a representative of three experiments. (B) Bar graph showing the mRNA expression of NCR1, FcεRIγ, and CD3ζ as determined by RT-PCR from purified NK cells isolated from naïve spleens of *Fcer1g*^+/–^ and *Fcer1g*^–/–^ mice (n = 3). (C) Intracellular expression of CD3ζ on splenic NK cells from naïve or i.v infected *Fcer1g*^+/–^ and *Fcer1g*^–/–^ mice with 2 x 10^4^ PFU of LCMV-Docile for 36 hours (n = 3–4). (D) Histogram depicting surface and intracellular staining of NKp46 on splenic NK cells from naïve or i.v infected *Fcer1g*^+/–^ and *Fcer1g*^–/–^ mice with 2 x 10^4^ PFU of LCMV-Docile for 36h (n = 4). The histograms are representative of two independent experiments. (E) Representative histogram for surface NKp46 expression on splenic NK cells from *Fcer1g*^+/–^ and *Fcer1g*^–/–^ mice treated *ex-vivo* with 20μg/ml MG-132 for 48 hours as indicated (n = 3) *(left panel)*. In the right panel, the shown is median fluorescence intensity (MFI) for the same experiment (n = 3). Data are shown as mean ± SEM. Significant differences between the two groups were detected by unpaired two-tailed *t*-tests and are indicated as follows: ns, not significant; * *p<*0.05; ** *p*<0. 01; *** *p*<0.001.

FcRγ is known to contribute to NKp46 signaling [22]; however, its role in NKp46 expression was unclear to us. To elucidate this role, we first determined whether Fc-receptor signaling by NK cells contributes to the upregulation of NKp46. Next, we tested the expression of NK cell activating receptors on splenic NK cells isolated from *Jh*^*–/–*^ mice which lack all serum immunoglobulins due absence of functional B cells and found that the absence of antibodies did not influence the expression of NKp46, a finding suggesting an immunoglobulin-FcR axis-independent role of FcRγ (Fig 3A). Strikingly, once we examined the expression of *NCR1* at mRNA level, we found normal expression of *NCR1* mRNA by NK cells in *Fcer1g*^–/–^ mice, similar to that found in WT mice (Fig 3B). This finding implies that the paucity of NKp46 expression in *Fcer1g*^–/–^ mice is not mediated by transcriptional inactivity of the *NCR1* gene. Also, the expression of CD3ζ mRNA, which is known to interact with NKp46 in a signaling complex at protein level [22], was similar in WT and *Fcer1g*^–/–^ mice (Fig 3B). Therefore, we proposed that FcRγ stabilizes NKp46 protein. Moreover, CD3ζ protein which is associated to FcRγ in transmembrane region and known to provide activation signaling downstream to NCR1/NKp46 receptor [22] was expressed at a high level in the absence of FcRγ (Fig 3C). In addition, intracellular staining of NKp46 showed no expression of this protein by *Fcer1g*^–/–^ NK cells which rule out the intracellular sequestration/internalization of NKp46 protein (Fig 3D). From these findings, we postulated that the presence of FcRγ in the endoplasmic reticulum (ER) and the Golgi apparatus may stabilize the NKp46 protein and prevents its degradation. In fact, treating NK cells with the proteasome inhibitor MG-132 enhanced the expression of NKp46 in splenic NK cells derived from *Fcer1g*^–/–^ mice to the level of NKp46 expression in WT (untreated) (Fig 3E). These data indicate that NKp46 protein is stabilized by FcRγ and absence of FcRγ in NK cells results in proteasomal degradation of NKp46 protein.

### FcεRIγ curtails CD8^+^ T-cell functions during chronic LCMV infection

**Fig 4:**
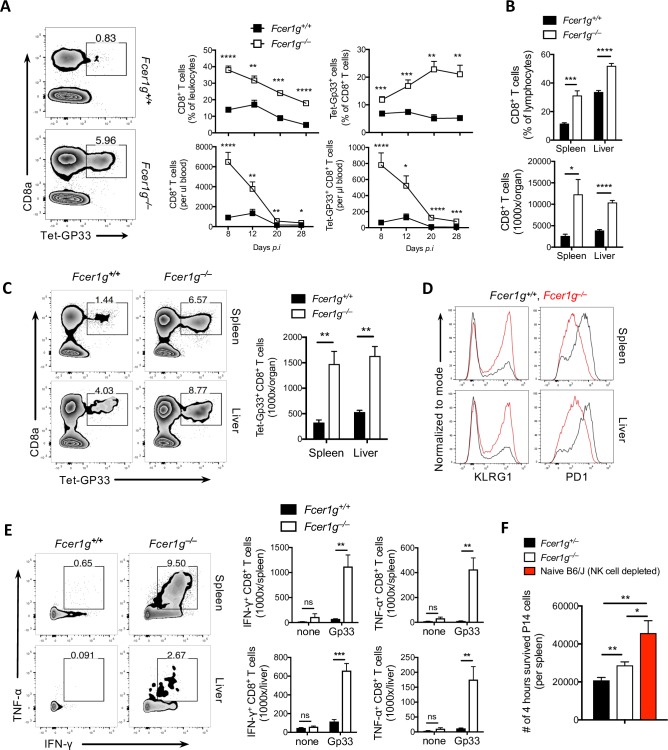
FcεRIγ curtails CD8^+^ T-cell functions during chronic LCMV infection. *Fcer1g*^+/+^ and *Fcer1g*^–/–^ mice were infected i.v. with 2 × 10^4^ PFU of LCMV-Docile and were bled at various time points or put to death on day 8 after infection. (A) The left representative FACS plot showing the frequency of glycoprotein (GP)33-Tet^+^ CD8^+^ T cells of total leukocytes in blood 8 days after infection. The right panel shows graphs of the kinetics for the frequency and number of CD8^+^ T cells (middle; n = 3–12) and virus-specific GP33-Tet^+^ CD8^+^ T cells in blood at the indicated time points (right; n = 3–12). Data are pooled from 3 independent experiments. (B) Frequency and total number of CD8^+^ T cells from spleen and liver on day 8 after infection (n = 4). (C) Representative FACS plots and graphs showing the frequency and total number of GP33-Tet^+^ CD8^+^ T cells in spleens and livers on day 8 after infection (n = 4). (D) Representative histogram showing the expression of PD1 and KLRG1 on GP33-Tet^+^ CD8^+^ T cells in spleens and livers on day 8 after infection (n = 4) (E) FACS plots (left panel) and graphs (right panel) depict the percentage and total numbers of CD8^+^ T cells producing interferon (IFN)-γ and tumor necrosis factor (TNF)-α in spleens and livers on day 8 after infection. The cells were stimulated *in-vitro* for 5 hours in the presence or absence of GP33 peptide (n = 4). (F) The bar graph represents the number of activated P14 cells after 4 hours of transfer in NK cells-depleted naïve C57BL6/J (B6/J) mice or *Fcer1g*^+/–^ and *Fcer1g*^–/–^ mice which were i.v infected with 200 PFU of LCMV-WE strain 3 days before the transfer (n = 6–7). Data are pooled from 2 independent experiments. The detailed protocol is described in materials and methods (*in-vivo* killer assay). Data are shown as mean ± SEM. Significant differences between the two groups were detected by unpaired two-tailed *t*-tests and are indicated as follows:: ns, not significant; * p<0.05; ** p<0. 01; *** p<0.001; **** p<0.0001.

To address the question of whether the lack of FcRγ indeed limits virus-specific CD8^+^ T-cell functions during chronic viral infection, we infected adult *Fcer1g*^+/+^ and *Fcer1g*^–/–^ mice with LCMV-Docile and determined CD8^+^ T-cell responses. The blood of *Fcer1g*^–/–^ mice exhibited enhanced CD8^+^ T-cell responses compared to *Fcer1g*^+/+^ littermates (Fig 4A). High number of LCMV-specific CD8^+^ T cells in the blood of *Fcer1g*^–/–^ mice were associated with enhanced percentages and numbers of CD8^+^ T cells in the spleen and liver at days 8 and 28 after infection (Fig 4B and 4C, S1A Fig). Interestingly, virus-specific CD8^+^ T cells in *Fcer1g*^–/–^ mice exhibited enhanced expression of KLRG1 and reduced expression of PD-1 (Fig 4D), a finding suggesting the functional and activated status of CD8^+^ T cells in *Fcer1g*^–/–^ mice. In line with these findings, we also detected enhanced frequencies and total numbers of IFN-γ–producing and TNF-α–producing CD8^+^ T cells in the spleen and liver of *Fcer1g*^–/–^ mice (Fig 4E, S1B Fig). Even after 55 days of LCMV chronic infection, we could see sustained potent CD8^+^T cells response in *Fcer1g*^–/–^ mice indicating the durable effect of FcεRIγ on regulating CD8^+^T cells (S1C and S1D Fig). Infection with LCMV-WE strain which is a model for acute infection, *Fcer1g*^–/–^ mice showed higher frequencies of CD8^+^ T cells but similar frequency of virus specific CD8^+^ T cells (S2 Fig) which may be due to differences in the activation status of NK cells during acute infection as compared to the robust one in chronic infection as described before [4].

Next, to address whether NK cells are behind elimination of virus-specific CD8^+^ T cells directly in FcεRIγ-dependent manner, we performed *in-vivo* killer assay. Intriguingly, the *in-vivo* activated P14 cells which were transferred into infected recipients (WT and *Fcer1g*^–/–^ mice) and NK cells-deficient naïve WT as a control, show numeric increase in *Fcer1g*^–/–^ mice as compared to WT after 4 hours of *in-vivo* incubation whereas the P14 dominated in control group (Fig 4F), revealing the prominent role of NK cells in attenuating CD8^+^ T cells response with aid of FcRγ. Taken together, these data demonstrate that the lack of FcRγ is beneficial for the expansion of virus-specific CD8^+^ T cells during chronic LCMV infection.

### FcεRIγ exacerbates viral control during the course of chronic LCMV infection

**Fig 5:**
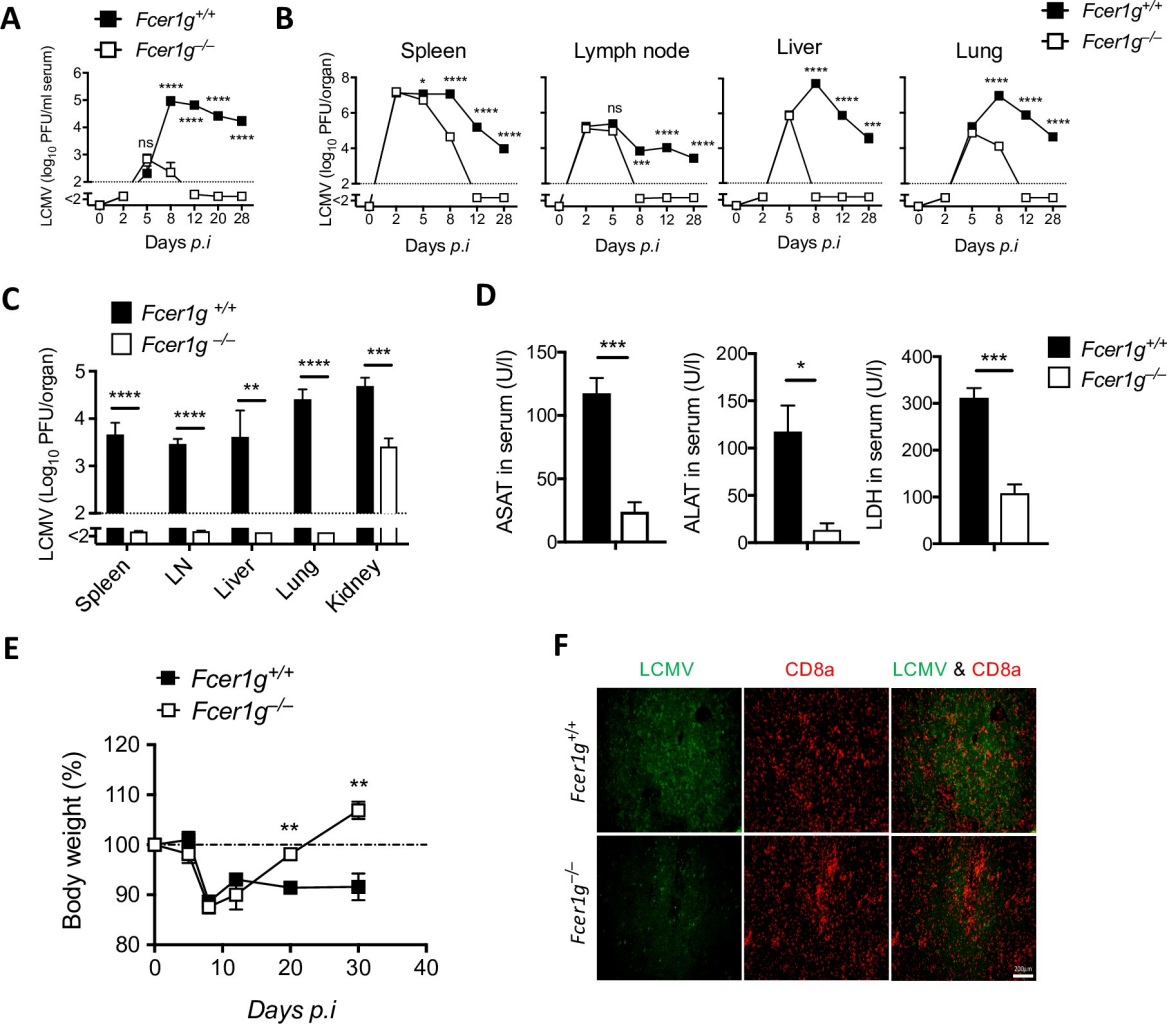
FcεRIγ exacerbates viral control during the course of chronic LCMV infection. Several groups of *Fcer1g*^+/+^ and *Fcer1g*^–/–^ mice were infected i.v with 2 × 10^4^ PFU of LCMV-Docile, were bled or killed at diverse time points, and were analyzed for certain variables. (A) Kinetics of viral titers in serum at the indicated time points after infection (n = 4–8). Data are pooled from 3 independent experiments. (B) Kinetics of viral titers in various organs at the indicated time points after infection (n = 3–4). (C) Viral titers in various organs on day 28 after infection (n = 7–8). Data are pooled from 2 independent experiments. (D) Levels of aspartate aminotransferase (AST), alanine aminotransferase (ALT), and lactate dehydrogenase (LDH) measured in serum on day 12 after infection (n = 4). (E) Percentage of body weight is shown at various days after infection (n = 5). (F) Representative immunofluorescence for liver histological sections from *Fcer1g*^+/–^ and *Fcer1g*^–/–^ mice stained for LCMV nucleoprotein (green) and CD8^+^ T cells (red) at day 12 after infection. One slide representative of 4 slides is shown. Scale bar, 200μm. Data are shown as mean ± SEM. Significant differences between the two groups were detected by unpaired two-tailed *t*-tests and are indicated as follows: ns, not significant; * *p<*0.05; ** *p*<0.01; *** *p*<0. 001; **** *p*<0.0001.

We explored the differences in the CD8^+^ T-cell response between WT and *Fcer1g*^–/–^ mice that may result in delayed virus control. This topic is of special interest because FcRγ may also be involved in functions that promote virus control. To gain insights, we infected WT and *Fcer1g*^–/–^ mice with 2 × 10^4^ plaque-forming units (PFU) of LCMV-Docile and analyzed virus control. The lack of FcRγ in *Fcer1g*^–/–^ mice led to rapid control of LCMV (Fig 5A–5C). LCMV was cleared from the circulation and from most of the organs in *Fcer1g*^–/–^ mice within 12 days with a limited existence in kidney (Fig 5A–5C). On contrary, higher viral titers were found in lymphatic and extra lymphatic organs of *Fcer1g*-sufficient mice (Fig 5A–5C) but almost resolved at long-term infection (S1E Fig). Moreover, WT mice showed severe immunopathology, whereas *Fcer1g*^–/–^ mice exhibited almost no liver pathology and only transient weight loss during persistent viral infection due to fast clearance of virus by robust virus-specific CD8^+^ T cell response (Fig 5D and 5E). Intriguingly, the liver damage in WT mice was due to high virus replication in liver cells that are target for LCMV-specific CD8^+^ T cell mediated killing (Fig 5F). These results suggest that lack of FcRγ result in faster control of persistent prone LCMV-infection with limited immunopathology.

### FcεRIγ on NK cells exerts an intrinsic effect on virus control

**Fig 6:**
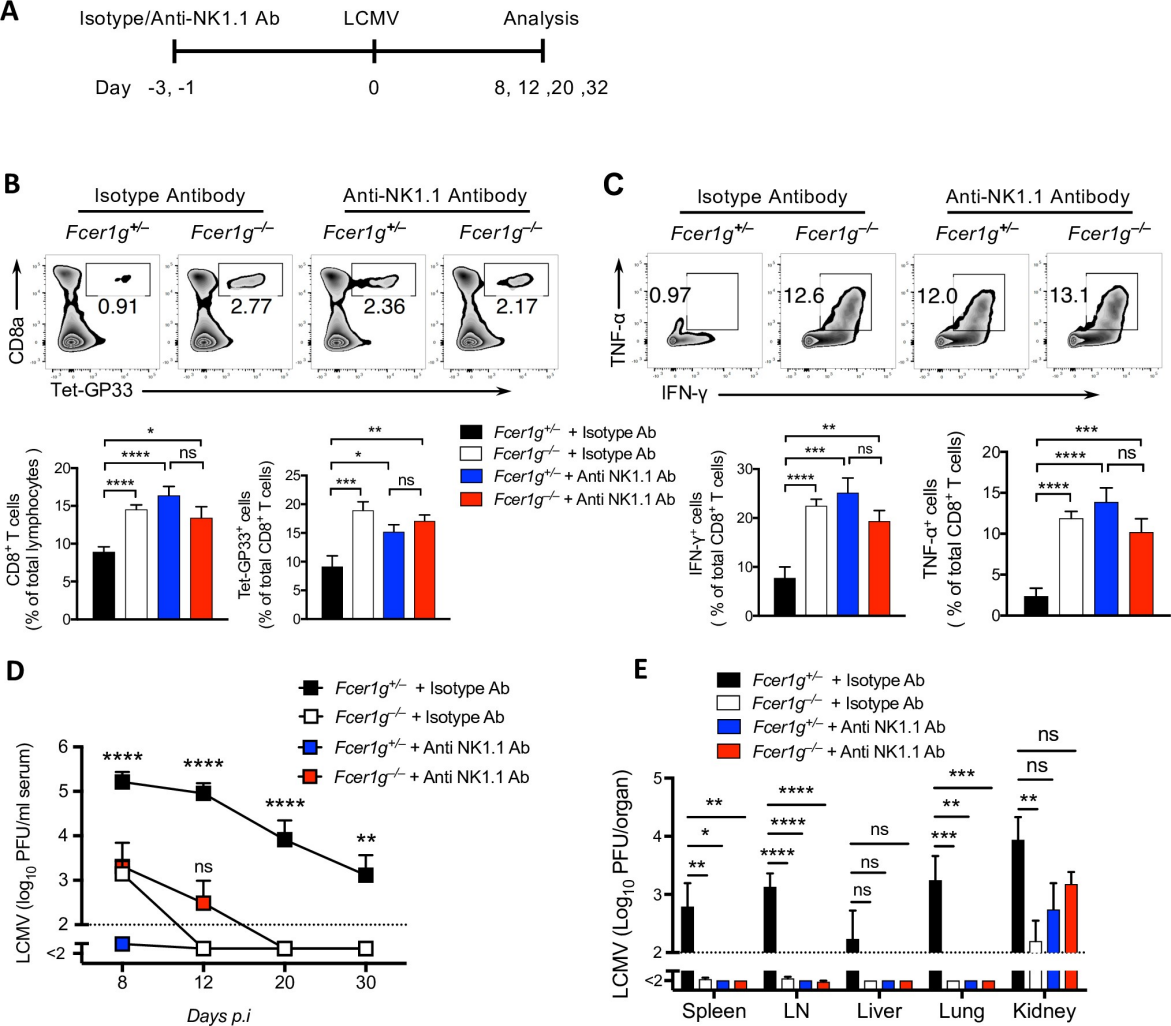
FcεRIγ of natural killer cells exerts an intrinsic effect on virus control. (A) Schematic of experimental setup. *Fcer1g*^+/–^ and *Fcer1g*^–/–^ mice were injected intraperitoneally with 200ug of anti-NK1.1 or isotype antibody on day -3 and day -1 and were infected i.v with 2 × 10^4^ PFU of LCMV-Docile at day 0. The mice were bled on days 8, 12, 20, and 32 after infection and were put to death on day 32 after infection. (B) The upper panel shows representative FACS plots for the frequency of glycoprotein (GP)33-Tet^+^ CD8^+^ T cells in the spleens on day 32 after infection. The lower panel shows graphs indicating the frequencies of CD8^+^ T cells and GP33-Tet^+^ CD8^+^ T cells in murine spleens on day 32 after infection (n = 6–10). (C) The FACS plots (upper panel) and graphs (lower panel) show the percentages of CD8^+^ T cells producing IFN-γ and TNF-α from splenocytes on day 32 after infection. These cells were stimulated *in-vitro* for 5 hours in the presence of GP33 peptide (n = 6–10). (D) Kinetics of viral titers in serum at indicated time points (n = 7–10). (E) Viral titers from various organs on day 32 after infection (n = 7–10). Data are pooled from two independent experiments (B-E). Data are shown as mean ± SEM. Significant differences between the groups were detected by unpaired two-tailed *t*-tests and are indicated as follows: ns, not significant; * *p<*0.05; ** *p*<0.01; *** *p*<0. 001; **** *p*<0.0001.

Next, we investigated whether the FcRγ-mediated regulatory NK cell functions explain the reduction in CD8^+^ T-cell responses and virus control in WT mice compared with *Fcer1g*^–/–^ mice. We depleted NK cells from WT and *Fcer1g*^–/–^ mice and infected the mice with LCMV-Docile (Fig 6A). NK cells ablation in WT mice brought the frequencies of functional virus-specific CD8^+^ T cells to the level found in *Fcer1g*^–/–^ mice which were treated with anti-NK1.1 or isotype antibody (Fig 6B and 6C). Virus control in NK cell–depleted WT mice was similar to that in *Fcer1g*^–/–^ mice treated with anti-NK1.1 or isotype antibody in most lymphoid, non-lymphoid organs as well as in the serum (Fig 6D and 6E), which suggest the pivotal role of FcεRIγ–deficient NK cells in honing the CD8^+^ T cells as well as resolving the persistent viral infection.

## Discussion

In the study described here, we delineated the paramount role of FcRγ in the NK cell–mediated regulation of virus-specific CD8^+^ T cells during chronic LCMV infection. Although it is well established that NK cells can curtail the CD8^+^ T-cell response, the identity of the molecules that are involved in modelling the regulatory capability of NK cells is still elusive. This study found that FcRγ plays an indispensable role in the NK cell–facilitated elimination of virus-specific CD8^+^ T cells and this effect is augmented by the lack of IFN-I signaling in CD8^+^ T cells, as described previously [9, 10]. A deficiency in FcRγ during chronic LCMV infection led to normal activation of NK cells but could not trigger cytotoxicity because of the lack of activating receptor NKp46. This lack of cytotoxicity resulted in a great degree of virus-specific CD8^+^ T-cell expansion and ultimately cleared persistent prone viral infection within 12 days with minimal immunopathology.

FcRγ is known to be involved in classical functions of NK cells, macrophages, neutrophils, basophils, and mast cells; these functions include antibody-dependent cell-mediated cytotoxicity (ADCC), phagocytosis and allergic reactions [30, 31]. Aside from expression of FcRγ on different immune cells (S3 Fig), we found that almost all NK cell express FcRγ but deficiency of FcRγ could not affect the development and activation of NK cells, a finding indicating that NK cell development and activation is independent of FcRγ signaling. We also found that the nonselective phase of NK cell activation, which is mainly dependent on innate immune cytokines [25, 26] and is indicated by high cell-surface expression of CD69, CD27, or KLRG1 and increased production of IFN-γ, granzyme B and perforin, is similar in FcεRIγ-sufficient and FcεRIγ-deficient NK cells.

The specific activation of NK cells is controlled by signaling of KARs or killer cell–inhibitory receptors [32, 33]. These receptors interact with ligands expressed on target cells, and the absolute balance in the signaling of KARs and killer cell–inhibitory receptors determines the fate of target cells. This study found that KARs such as natural killer group 2, member D (NKG2D), stem cell antigen-1 (Sca-1), and Ly49H are not affected but the expression of NKp46 is completely missing on NK cells in the absence of FcRγ. Furthermore, we showed that FcεRIγ-deficient NK cells exhibit reduced levels of TRAIL and PKCθ. TRAIL is known to induce cell death by binding to its receptor on target cells [27], a finding indicating that FcεRIγ-deficient NK cells are less efficient than FcεRIγ-sufficient NK cells in the direct induction of apoptosis. This finding is similar to those of a recently published study, which found that NKp46-deficient NK cells lack surface expression of TRAIL [34]. PKCθ is involved in signaling downstream of KARs that induces the activation of NK cells [35]. This finding suggests that KAR-induced activation is reduced in FcεRIγ-deficient NK cells, but this requires further study for clarification.

In contrast to previous study which revealed that NKp46 expression is dependent on FcRγ and CD3ζ [36], we found high expression of CD3ζ in the absence of FcRγ, a finding indicating that NKp46 expression is independent of CD3ζ. NKp46, FcRγ and CD3ζ are present as a signaling complex on NK cells [22]. The absence of CD3ζ from NK cells was reported to be correlated with high expression of CD16 receptors [37], this increased expression could be a compensatory mechanism providing proper downstream signaling to the NKp46/FcRγ/CD3ζ complex. We demonstrated that high expression of CD3ζ in the absence of FcRγ could also fine-tune appropriate signaling, but this finding must be validated by further studies.

The current study found a complete lack of NKp46 in FcεRIγ-deficient NK cells, which would affect the natural killing activity induced by NKp46 signaling. FcεRIγ-induced expression of NKp46 by NK cells was independent of the cross-linking of Fc receptors (CD16) with antibodies, because we found no alteration of NKp46 expression by NK cells in serum immunoglobulins-deficient mice. The transcriptional activity of *NCR1* was unchanged; mRNA levels were similar in the absence and presence of FcεRIγ. However, intracellular and surface NKp46 level were completely missing in *Fcer1g*^*–/–*^ mice, a finding indicating a higher level of protein degradation and instability in the absence of FcεRIγ rather than protein sequestration. Indeed, we could rescue NKp46 protein degradation by inhibiting the proteasomal activity of NK cells, after *ex-vivo* treatment with MG-132. From this study the mechanism remained to be investigated that how exactly the FcεRIγ expression stabilizes the NKp46 protein and prevents its degradation by proteasome.

During viral infection, NK cells target CD8^+^ T cells and promote viral persistence and immunopathology [5]. Using a chronic LCMV model, we found that FcRγ expression by NK cells inhibits virus-specific CD8^+^ T-cell responses. In *Fcer1g*^*–/–*^ mice, where the LCMV-specific CD8^+^ T cells show potent response, the cell surface phenotype of virus-specific CD8^+^ T cells was found to be similar to that seen during acute infection [38]. This finding suggests that FcRγ not only activates NK cell cytotoxic functions but also leads to CD8^+^ T-cell exhaustion. Thus, FcεRIγ deficiency imparts an acute signature mimicry for the LCMV chronically infected mice. Nevertheless the impact of FcεRIγ in CD8^+^ T-cell exhaustion requires further studies. A recent study showed that PD-L1 expressing type 1 innate lymphoid cells (ILC1s; authors named this cell type as liver resident NK cells) inhibit T cell functions during LCMV infection in liver microenvironment [39]. Conventional NK (cNK) cells and ILC1s belong to group 1 ILCs and both equally express NKp46. ILC1s are described to lack cytolytic activity like cNK cells [40] and express higher levels of negative regulators of immune response such as PD-L1, CD39 or CD73 [39]. In the liver group 1 ILCs comprise ILC1s and cNK cells while spleen contains only cNK cells [40]. In this report we focused on the regulatory function of splenic NK cells. However, it is not known and remained to be investigated, how FcRγ regulate the functions of ILC1s and cNK cells in the liver which might influence the CD8^+^ T cell response during chronic virus infection.

The FcRγ molecule is also part of a TCR:CD3 complex[41]. The question of whether FcRγ intrinsically affects T-cell functions cannot be underestimated. During acute viral infection the regulatory role of NK cells are revealed to be negligible [4]. In acute infection model of LCMV, we found significantly reduced frequencies of total CD8^+^ T cell in WT compared to *Fcer1g*^–/–^ mice but no difference in virus specific CD8^+^ T cell frequencies is observed (S2 Fig) which hints T cell intrinsic role of FcRγ. However, more studies are needed to clarify this phenotype. Here, we focused to study the role of FcRγ in term of regulating the CD8^+^ T cell function by NK cells, it cannot be ignored that FcRγ dependent functions of NK cells may regulate or kill dendritic cells (DCs) which in turn influence the CD8^+^ T cell response. Indeed, we found higher level of TRAIL expression on WT as compared to *Fcer1g*^*–/–*^ NK cells which is shown before that NK cells kill DCs in TRAIL dependent manner [42] but further investigation is needed to provide the clue for involvement of FcRγ in this activity.

In conclusion, we have shed some light on the FcεRIγ molecule as a regulator of NK cell–dependent CD8^+^ T-cell functions during chronic viral infection. Moreover, FcεRIγ protects proteasomal degradation of NCR1 protein, thereby stabilizing the intracellular and surface expression of NCR1. Furthermore, FcεRIγ exerts a negative impact on virus-specific CD8^+^ T cells; this impact results in T-cell exhaustion, a common phenomenon of various fulminant chronic viral infections such as HBV, HCV, or HIV, as well as certain tumors. Like other molecules, such as programmed cell death protein 1 (PD-1), FcεRIγ (or one of its downstream signaling molecules) could be a target of a therapeutic regimen. It may offer a new therapeutic avenue for reinvigorating T-cell responses and could be extended to other immune cells, thereby leading to better outcomes. Such other options remain to be investigated.

## Materials and methods

### Ethics statement

Animal experiments were authorized by the Veterinäramt Nordrhein-Westfalen (Düsseldorf-Essen, Germany) and were conducted in accordance with the German laws for animal protection or according to institutional guidelines at the Ontario Cancer Institute of the University of Toronto Health Network. All animal experiments were approved under license number 84-02-04.2013.A242 by Landesamt für Natur, Umwelt und Verbraucherschutz Nordrhein-Westfalen. All animal care and use protocols adhere to national (Tierschutzgesetz) and European (Directive 2010/63/EU) laws and regulations as well as European Federation of Animal Science Associations (FELASA) http://www.felasa.eu/. Animals were euthanized using the cervical dislocation method.

### Mice

*Fcer1g*^*–/–*^ mice were purchased from Jackson Laboratory (Bar Harbor, ME, USA; stock# 002847) and were maintained on a mixed (B6; 129P2) background. Littermate WT or heterozygous mice (*Fcer1*^*+/*–^) served as controls for all experiments. *Jh*^*–/–*^ and P14/CD45.1 mice were originally obtained from Tak W. Mak (The Campbell Family Cancer Research Institute and University Health Network, Toronto, Canada). *Jh*^*–/–*^ lack the gene for heavy chain joining region, hence do not develop functional B cells and serum immunoglobulins. *Jh*^*–/–*^ mice used in the current study were maintained on the C57BL/6 genetic background. P14/CD45.1 mice, which have LCMV glycoprotein (Gp)33-41–specific TCR as a transgene and *IFNAR*^*–/–*^ *× P14/CD90*.*1* (generously provided by Prof. Philipp A Lang; senior author in this paper) were used for adoptive transfer experiments and were maintained on a C57BL/6 background. All mice were matched in sex, age, and weight to control mice. All of the mice used in this study were 6 to 12 weeks old and were housed in single ventilated cages. Animals exhibiting severe symptoms of sickness or substantial weight loss during LCMV infection were considered dead for statistical analyses.

### Virus and plaque assay

The LCMV-WE strain was originally obtained from F. Lehmann-Grube (Heinrich Pette Institute, Hamburg, Germany) and was propagated on L929 cells (obtained from ATCC, NCTC clone 929). LCMV-Docile was provided by Prof. Dr. R. Zinkernagel (University of Zurich, Zurich, Switzerland) and was propagated on L929 cells. For this study all mice were infected intravenously with a dose described in figure legends according to the experiment. Mice were put to death at different time as mentioned in the figure legends, and LCMV viral titers were detected on MC57 fibroblasts (provided by the Ontario Cancer Institute (Toronto, ON, Canada)) by a plaque-forming assay, as previously described [43]. Briefly, organs were smashed in Dulbecco’s modified Eagle medium (DMEM) containing 2% fetal calf serum (FCS), titrated 1:3 over 12 steps in 96-well round-bottom plates, and plaqued onto MC57 cells. After incubation for 3 h at 37°C, an overlay was added (1:1 mixture of methyl cellulose and Iscove’s Modified Dulbecco’s Medium), and the virus preparation was again incubated at 37°C. Plaques were stained 48 hours later. For staining, cells were fixed with 4% formaldehyde solution in phosphate-buffered saline (PBS), permeabilized with a 1% Triton-X solution in PBS, blocked with 10% FCS in PBS, and stained with anti-LCMV nucleoprotein (NP) antibody (made in house). Enhanced chemiluminescence (ECL)-conjugated anti-rabbit IgG antibody was used as a secondary antibody. Plaques were detected by color reaction (0.2 M Na_2_HPO_4_ + 0.1 M citric acid + 30% H_2_O_2_ + o-phenylenediamine dihydrochloride). All chemicals were purchased from Sigma-Aldrich (Germany).

### Antibodies, tetramer, cell-staining, and flow cytometry analysis

All of the antibodies used in this study are listed in S1 Table. The LCMV-specific CD8^+^ T-cell response upon LCMV infection was measured with a tetramer complex of major histocompatibility complex (MHC) class I (H-2D^b^) and LCMV GP_33-41_ (KAVYNFATM) peptide. Tetramers were provided by the National Institutes of Health (NIH) Tetramer Facility (Bethdsda, MD, USA). Cells were stained with allophycocyanin (APC)-labeled GP33 MHC class I tetramer (GP33/H-2D^b^) for 15 minutes at 37°C. After incubation, the samples were stained with monoclonal antibody to CD8a for 30 minutes at 4°C. Absolute numbers of GP33-specific CD8^+^ T cells were counted by fluorescence-activated cell sorting (FACS) with calibrating beads (340486; BD Bioscience, Germany). For Lamp-1 staining, anti-CD107a antibody was added for the 5-hr incubation period, and the immunofluorescence was measured after additional staining with anti-NK1.1 antibody. For intracellular cytokine staining, smashed splenocytes from LCMV-infected mice were cultured in 5% FCS DMEM medium supplemented with LCMV GP_33-41_ peptide (5 μg/ml) for 1 hour at 37°C in an incubator. After 1 hour, brefeldin A (25 μg/ml; B7651; Sigma, Germany) was added, and the cells were incubated for another 4 hours at 37°C. After a total of 5 hours, splenocytes were washed with FACS buffer, stained for surface anti-mouse CD8 antibody at 4°C for 30 minutes and then fixed with 2% formalin in PBS at room temperature for 10 minutes. After another washing step, cells were incubated for intracellular staining with antibodies to IFN-γ and TNF-α in 0.1% saponin (S4521; Sigma) in FACS buffer for 30 minutes at 4°C, washed, and analyzed with flow cytometry.

### RNA isolation and quantitative reverse transcription polymerase chain reaction

Total RNA was isolated from NK cells with the RNeasy Mini Kit (Qiagen, Hilden, Germany). For RNA quantification, a NanoDrop ND-1000 spectrophotometer (Peqlab Biotechnologie GmbH, Erlangen, Germany) was used. Complementary DNA synthesis was performed with the QuantiTect Reverse Transcription Kit *(*Qiagen). For quantitative reverse transcription polymerase chain reaction (qRT-PCR) either the Fast SYBR Green Master Mix (Applied Biosystems, Darmstadt, Germany) or the TaqMan Fast Universal PCR Master Mix (2X; Applied Biosystems) was used on a 7500 Fast Real-Time PCR System (Applied Biosystems, Darmstadt, Germany). Primers for *NCR1*, *Fcer1g and CD247* were purchased from Qiagen or Thermofisher (Germany). For analysis, the expression levels of all target genes were normalized against glyceraldehyde 3-phosphate dehydrogenase (GAPDH; ΔCt). Gene expression values were calculated with the ΔΔCt method.

### *In vivo* killer assay

10^6^ negatively MACS sorted CD8^+^ T cells from spleens of P14 x CD45.1 mice were adoptively transferred into *Fcer1g*^*–/–*^ mice and on next day, those mice were infected i.v. with 200 PFU of LCMV-WE strain. After 5 days, negatively MACS sorted total CD8^+^ T cells (1 x 10^6^ cells per mouse) were transferred into NK cell depleted naïve C57BL/6 mice or WT and *Fcer1g*^*–/–*^ mice which were already i.v infected with 200 PFU of LCMV-WE 3 days before. After 4 hours of *in-vivo* incubation in recipient mice, spleens were harvested and the total number of P14 cells were analyzed and calculated by FACS.

### Lymphocyte adoptive transfer

Splenocytes or negatively sorted CD8^+^ T cells from P14/CD45.1 or *IFNAR*^*–/–*^ *× WT/CD90*.*1* mice were injected intravenously into mice of interest. One day later, mice were infected with LCMV-docile strain. To identify the proliferation of transferred cells, splenocytes from P14/CD45.1 mice were stained with 1 μM carboxyfluorescein succinimidyl ester (CFSE; Invitrogen, Germany) in PBS for 10 minutes at 37°C, washed 2 times with 10% FCS DMEM media, suspended in plain DMEM media and injected intravenously into mice. One day later, mice were infected with LCMV-WE and the proliferation of P14 T cells in the spleen was assessed with CFSE dilution by flow cytometry.

### Liver enzyme activity measurements

The activity of alanine aminotransferase (ALT), aspartate aminotransferase (AST), and lactate dehydrogenase (LDH) was measured in the Central Laboratory, University Hospital Essen, Germany.

### Purification and culture of NK cells

NK cells were negatively sorted with a mouse NK cell isolation kit (130-115-818; Milteny Biotec, Germany) according to the manufacturer’s protocol. For NK cultures, sorted NK cells were stimulated with 1,000 U/ml IL-2 (Miltenyi) for 2 days and were then treated with 20μg/ml of MG-132 (a proteasome inhibitor) purchased from (Enzo Life Sciences, Farmingdale, NY, USA; BML-Pl102-0025).

### NK cell depletion

NK cells were depleted with an intraperitoneal injection of anti-NK1.1 (clone PK136 from Bioxcell; 200 μg per mouse) or mouse IgG2a isotype control (from Bioxcell) on day 3 before infection and on day one after infection, as previously described [5].

### Histology

Histologic analyses of snap-frozen tissue were performed with a mAb to anti-CD8a-PE, and mouse monoclonal antibodies to LCMV nucleoprotein (NP; made in house). In short, sections were fixed with acetone for 10 min, and nonspecific antigen binding was blocked in PBS containing 2% FCS for 15 min, followed by staining with various antibodies for 45 min. All antibodies were diluted 1:100 from their original concentration in blocking solution. Images of stained sections were acquired with a fluorescence microscope (KEYENCE BZ II analyzer; KEYENCE Corporation of America, Itasca, IL, USA).

### Statistical analysis

Data are depicted as means ± S.E.M. Unpaired Student’s *t*-tests were used to detect statistically significant differences between groups. *P* values lower than 0.05 were considered statistically significant. Statistical analyses and graphical presentations were computed with Graph Pad Prism, version 7.03 (GraphPad Software,USA).

## Supporting information

S1 Fig FcεRIγ deteriorates CD8^+^ T cell response during chronic LCMV infection. *Fcer1g*^*+/+*^ and *Fcer1g*^*–/–*^ mice were infected intravenously with 2 x 10^4^ PFU of LCMV-Docile, sacrificed at day 28 (A-B) or day 55 (C-D), and analyzed for different parameters in the spleen. **(A)** Representative FACS plots show Tet-GP33^+^ CD8^+^ T cells and Tet-NP396^+^ CD8^+^ T cells (left panel). The right panel shows the frequency of CD8^+^ T cells and the total number of Gp33-Tet^+^ CD8^+^ T cells (n = 3–4). **(B)** The FACS plots (left panel) and graphs (right panel) depict the percentage and the total number of CD8^+^ T cells which were positive for IFN-γ and TNF-α. These cells were stimulated *in-vitro* for 5 hours in the presence or absence of GP33 or NP396 peptide (n = 3–4). **(C)** The graphs represent the frequency of Gp33-Tet^+^ CD8^+^ T cells in blood and spleen (n = 5). **(D)** In the left panel, representative FACS plots depict the percentage IFN-γ and TNF-α positive cells of total CD8^+^ T cells in the spleens. In right panel, the bar graph depicts the frequency of IFN-γ and TNF-α positive CD8^+^ T cells. These cells were stimulated *in-vitro* for 5 hours in the presence or absence of GP33 peptide (n = 5). **(E)** The bar graph shows the viral titers from different lymphoid and non-lymphoid organs (n = 5). Data are shown as mean ± SEM. Significant differences between the groups were detected with unpaired two-tailed *t*-tests and are indicated as follows: NS, not significant; * *p<*0.05; ** *p*<0.01; *** *p*<0.001; **** *p*<0.0001.
(TIF)

S2 Fig FcεRIγ demotes CD8^+^ T cell response during acute LCMV infection. *Fcer1g*^*+/+*^ and *Fcer1g*^*–/–*^ mice were infected i.v. with 200 PFU of LCMV-WE, sacrificed at day 8 and analyzed for different parameters. **(A)** Representative FACS plots show GP33-Tet+ CD8+ T cell frequency in blood (left panel) and spleen (right panel) (n = 4). Graphs show the frequency and absolute number of CD8+ T cells and Gp33-Tet+ CD8+ T cells in the blood **(B)** and spleen **(C)** (n = 4). Data are shown as mean ± SEM. Significant differences between the groups were detected with unpaired two-tailed t-tests and are indicated as follows: NS, not significant; *p<0.05; **p<0.01; ***p<0.001.
(TIF)

S3 Fig FcεRIγ is widely expressed on different immune cells. Representative histogram for the intracellular staining of FcεRIγ on different naïve splenic innate and adaptive immune cells with *(n = 4*).
(TIF)

S1 Table List for detailed information of antibodies used in this study. (PDF)
