# Supplementary figures and images for: NK cell–intrinsic FcεRIγ limits CD8+ T-cell expansion and thereby turns an acute into a chronic viral infection
Source: PLoS Pathog. 2019 Jun 20;15(6):e1007797. doi: 10.1371/journal.ppat.1007797 (PMC6605677; doi:10.1371/journal.ppat.1007797)

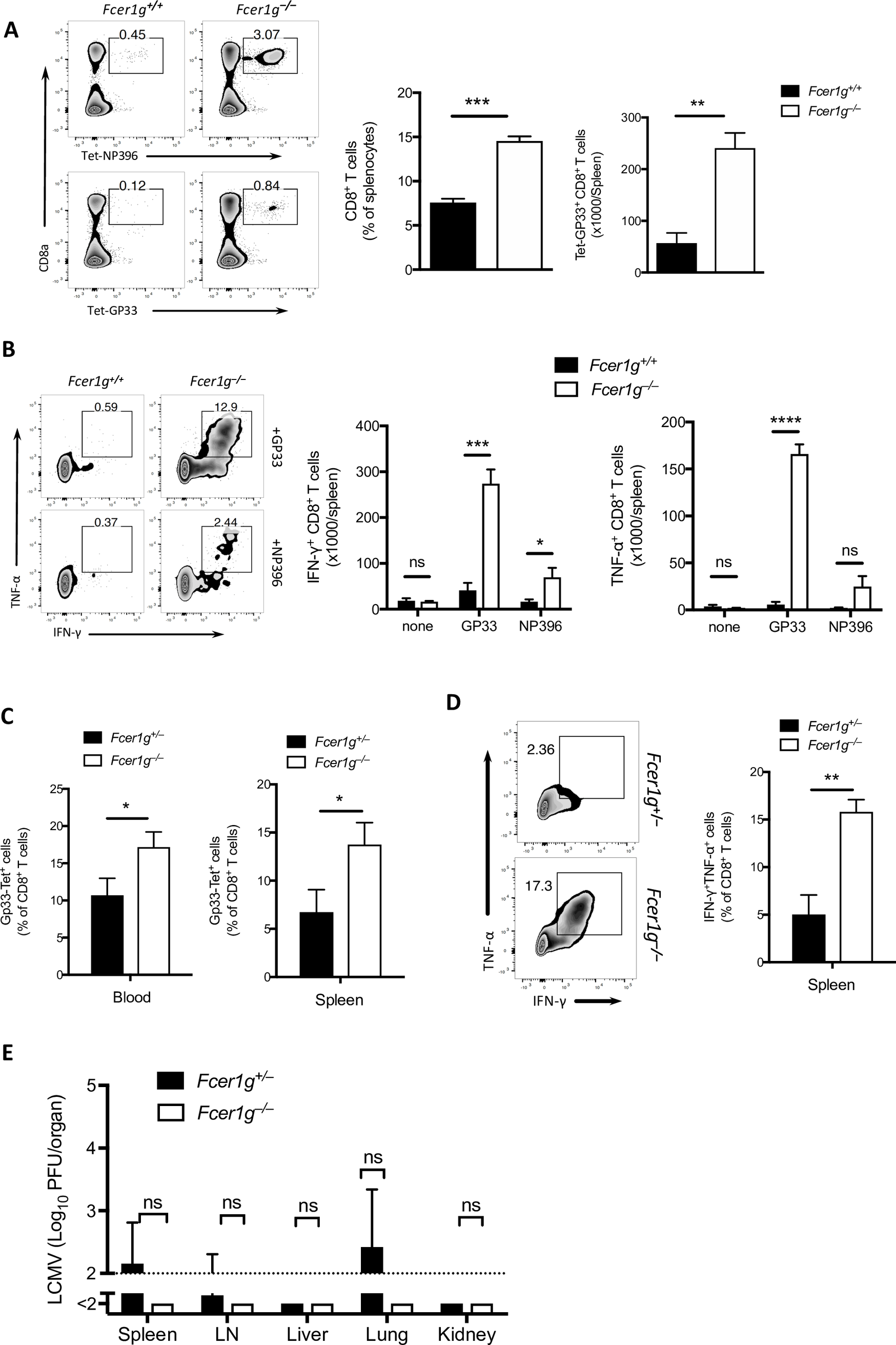

Supplement: S1 Fig — Fcer1g+/+ and Fcer1g–/– mice were infected intravenously with 2 x 10^4^ PFU of LCMV-Docile, sacrificed at day 28 (A-B) or day 55 (C-D), and analyzed for different parameters in the spleen. (A) Representative FACS plots show Tet-GP33+ CD8+ T cells and Tet-NP396+ CD8+ T cells (left panel). The right panel shows the frequency of CD8+ T cells and the total number of Gp33-Tet+ CD8+ T cells (n = 3–4). (B) The FACS plots (left panel) and graphs (right panel) depict the percentage and the total number of CD8+ T cells which were positive for IFN-γ and TNF-α. These cells were stimulated in-vitro for 5 hours in the presence or absence of GP33 or NP396 peptide (n = 3–4). (C) The graphs represent the frequency of Gp33-Tet+ CD8+ T cells in blood and spleen (n = 5). (D) In the left panel, representative FACS plots depict the percentage IFN-γ and TNF-α positive cells of total CD8+ T cells in the spleens. In right panel, the bar graph depicts the frequency of IFN-γ and TNF-α positive CD8+ T cells. These cells were stimulated in-vitro for 5 hours in the presence or absence of GP33 peptide (n = 5). (E) The bar graph shows the viral titers from different lymphoid and non-lymphoid organs (n = 5). Data are shown as mean ± SEM. Significant differences between the groups were detected with unpaired two-tailed t-tests and are indicated as follows: NS, not significant; * p<0.05; ** p<0.01; *** p<0.001; **** p<0.0001. (TIF) [file ppat.1007797.s001.tif]

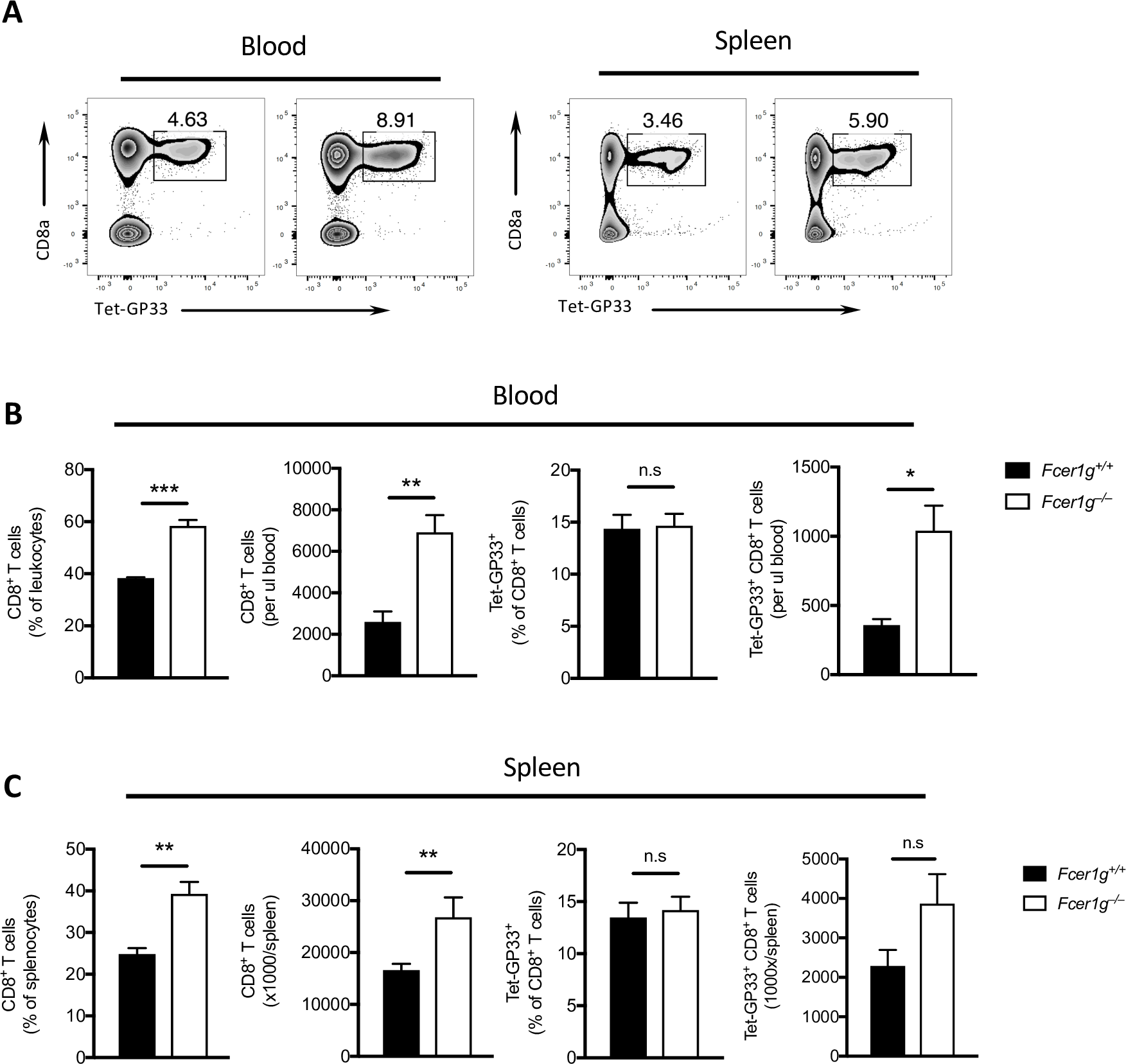

Supplement: S2 Fig — Fcer1g+/+ and Fcer1g–/– mice were infected i.v. with 200 PFU of LCMV-WE, sacrificed at day 8 and analyzed for different parameters. (A) Representative FACS plots show GP33-Tet+ CD8+ T cell frequency in blood (left panel) and spleen (right panel) (n = 4). Graphs show the frequency and absolute number of CD8+ T cells and Gp33-Tet+ CD8+ T cells in the blood (B) and spleen (C) (n = 4). Data are shown as mean ± SEM. Significant differences between the groups were detected with unpaired two-tailed t-tests and are indicated as follows: NS, not significant; *p<0.05; **p<0.01; ***p<0.001. (TIF) [file ppat.1007797.s002.tif]

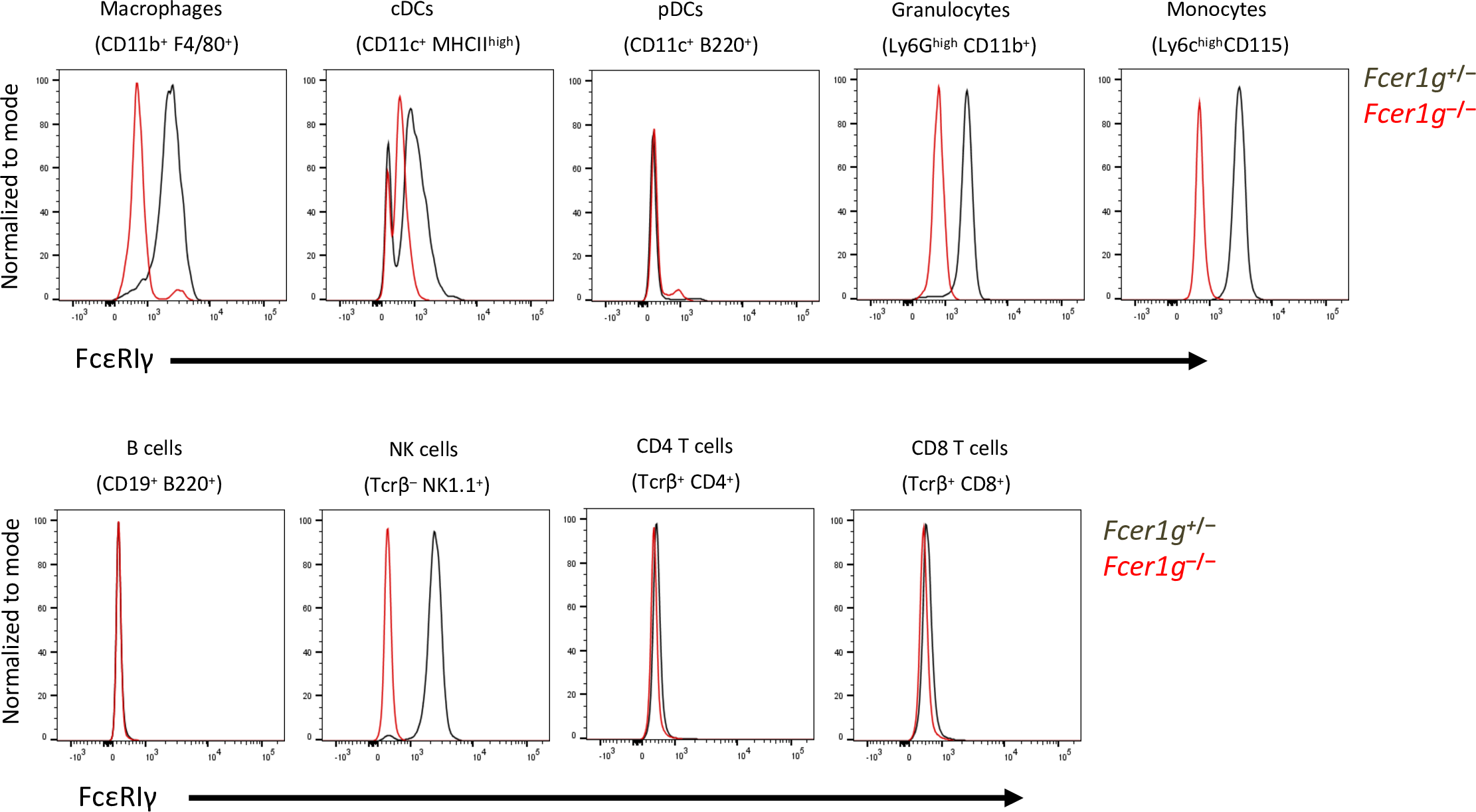

Supplement: S3 Fig — Representative histogram for the intracellular staining of FcεRIγ on different naïve splenic innate and adaptive immune cells with (n = 4). (TIF) [file ppat.1007797.s003.tif]
