# Supplementary material for: NK cell–intrinsic FcεRIγ limits CD8+ T-cell expansion and thereby turns an acute into a chronic viral infection
Source: PLoS Pathog. 2019 Jun 20;15(6):e1007797. doi: 10.1371/journal.ppat.1007797 (PMC6605677; doi:10.1371/journal.ppat.1007797)
Supplement: S1 Table — (PDF) [file ppat.1007797.s004.pdf]

Antibody	Conjugate	Clone	Company
TCR-β monoclonal antibody	PE	H57-597	eBioscience
Anti-mouse NK1.1	APC	PK136	Biolegend
Anti-mouse CD335 (NKp46) antibody	PE/Cy7	29A1.4	eBioscience
Anti-CD3ζ antibody	FITC	H146-968	Abcam
Anti-FcεRI antibody, γ subunit	FITC	polyclonal	Merck
Anti-mouse CD335 (NKp46) antibody	PE	29A1.4	Biolegend
Anti-mouse CD253 (TRAIL) antibody	PE/Cy7	N2B2	Biolegend
Perforin monoclonal antibody	PE	EBioOMAK-D	eBioscience
Ly-49H monoclonal antibody	FITC	3D10	eBioscience
Anti-human/mouse granzyme B antibody	Pacific Blue	GB11	Biolegend
Anti-mouse Ly-6A/E (Sca-1) antibody	APC/Cy7	D7	Biolegend
FITC Armenian hamster IgG isotype ctrl antibody	FITC	HTK888	Biolegend
Rat IgG2a, κ isotype ctrl antibody	PE/Cy7	RTK2758	Biolegend
Rat IgG2a κ isotype control	PE	eBR2a	eBioscience
Anti-mouse CD314 (NKG2D) antibody	FITC	CD314	Biolegend
Anti-mouse CD107a (LAMP-1) antibody	FITC	1D4B	Biolegend
IFN-γ monoclonal antibody	PE	XMG1.2	eBioscience
TNF-α monoclonal antibody	APC	MP6-XT22	eBioscience
CD69 monoclonal antibody (H1.2F3)	PE	H1.2F3	eBioscience
CD279 (PD-1) monoclonal antibody	FITC	RMP1-30	eBioscience
KLRG1 monoclonal antibody	PE-eFluor 610	2F1	eBioscience
CD27 monoclonal antibody	APC-eFluor 780	CXCR3-173	eBioscience
Anti-PKCθ antibody	-----	2F1	Abcam
AffiniPure goat anti-rabbit IgG (H+L)	FITC	LG.7F9	Jackson ImmunoResearch

CD90.1 (Thy-1.1) monoclonal antibody	APC	Polyclonal	eBioscience
CD45.1	PE	A20	ebioscience
CD8a	PE/Cy7	52-6.7	invivogen
CD45.2	V500	104	BD bioscience
CD4	APC	GK1.5	eBioscience
CD11b	Q-dot	M1/70	BD bioscience
F4/80	APC	BM8	eBioscience
Ly6G	PerCP/Cy5.5	RB6-8C5	invivogen
Ly6C	Pacific blue	HK1.4	eBioscience
CD115	PE	AFS98	eBioscience
B220	APC	RA3-6B2	eBioscience
CD19	Q-dot	1D3	eBioscience
CD11c	PE/Cy7	N418	eBioscience
MHC II	PE	M5/114.15.2	eBioscience

Abbreviations: APC, antigen-presenting cell; FITC, fluorescein isothiocyanate; PerCP, Peridinin-chlorophyll proteins ; IgG, immunoglobulin G; PE, phycoerythrin; TCR, T-cell receptor; TRAIL, TNF-related apoptosis-inducing ligand; PKC, protein kinase C theta; PE-Cy7: Phycoerythrin-Cyanine7.
